# Crystals structures of carbonate phases with Mg in Triassic rocks, mineral formation and transitions

**DOI:** 10.1038/s41598-023-46013-2

**Published:** 2023-10-31

**Authors:** Katarzyna J. Stanienda-Pilecki

**Affiliations:** Department of Applied Geology, Silesian University of Technology, Akademicka 2 Street, 44-100 Gliwice, Poland

**Keywords:** Solid Earth sciences, Space physics, Materials science

## Abstract

Detailed data on carbonate minerals different in magnesium content including their crystal structures were presented in this article. The samples were collected from the formations of the Lower Muschelkalk and the Upper Muschelkalk. X-ray diffraction and electron microprobe analysis were used for carbonate phases determination. The following minerals with different Mg content were identified: low-Mg calcite, high-Mg calcite, proto-dolomite, ordered dolomite and de-huntite. The content of Mg in high-Mg calcite is higher than in low-Mg calcite but lower than that of proto-dolomite. Proto-dolomite is characterized by lower content of Mg than typical for stoichiometric value for dolomite—ordered dolomite. Due to the reduced Mg content in identified huntite, this carbonate phase was named as de-huntite. The research results are significant because they are a source of new data on carbonate phases with different Mg content which build studied Triassic rocks and crystal structures of these minerals.

## Introduction

Carbonate minerals (MCO_3_), including mainly calcite phases, [CaCO_3_], dolomite phases [CaMg(CO_3_)_2_], huntite [CaMg_3_[CO_3_]_4_], magnesite [MgCO_3_] and others are widely distributed in different geological formations. The proper determination of elemental content of carbonate minerals is very important in earth science. X-ray diffraction and electron probe microanalysis (X-Ray microprobe analysis—EPMA) are the most commonly used analytical methods for the determination of elements in solid materials and have been widely utilized in geological research^1–4^.

The aim of the research was to identify carbonate phases varied in terms of magnesium content on the basis of X-ray diffraction and electron microprobe analysis (X-ray spectral microanalysis, microprobe measurements, EPMA). The carbonates’ phases transitions during sedimentary and diagenetic processes was also studied. Magnesium is one of the components of five determined carbonate phases: low-Mg calcite (low magnesium calcite), high-Mg calcite (high magnesium calcite, magnesio-calcite) proto-dolomite, ordered dolomite and huntite. X-ray diffraction allows to identify mineral phases on the basis of their crystal structures. With the increase of Mg in the crystal, the values of the diffraction lines decrease. It gives the possibility to identify carbonate phases with different Mg content. X-ray microprobe analysis allow to measure the element content in selected points of sample micro-area. This gives the possibility of precise measurement of the element content in a point. Precise measurement of magnesium allows to establish the possible carbonate phase in point. It was the reason for choosing this method to determine the type of carbonate phases with different Mg content in the analysed limestones.

The studied carbonate phases occur in Triassic limestones of the Opole Silesia^5–13^ and Triassic dolomites of the Upper Silesia^14–16^. Triassic limestones from the area of Polish part of Germanic Basin (South-West part of Poland—Opole Silesia) are the sediments of the Eastern part of this epicontinental basin. This area is the East zone of the Central Europe Triassic intracratonic basin which sequences of other parts are on the terrains of Germany, Netherlands, Slovakia, Hungary, Austria, Italy and Switzerland^14–16^.

During previous studies five carbonate phases varied in magnesium content: low-Mg calcite, high-Mg calcite, proto-dolomite, ordered dolomite and huntite were identified in the Triassic rocks of the Polish part of epicontinental Germanic Basin on the basis of X-Ray Diffraction and Electron microprobe analysis^5–13^. They occur in limestones (Lower Muschelkalk sediments of Gogolin, Górażdże, Terebratula and Karchowice Units- Opole Silesia)^5–13^ and dolomites (Upper Muschelkalk sediments of Tarnowice Unit- Upper Silesia)^14–16^. However, the crystal structures and chemical formulas of the mineral phases have not been studied in detail. Then this study is essential because the test results are a source of new data on the range of magnesium content in the analyzed carbonate phases. Moreover on the basis of the results it was possible to create the probable crystal structures of carbonate phases. The results of study also allow to understand the carbonates’ phases transitions during sedimentary processes and diagenesis.

Researches of carbonate phases varied in terms of magnesium content are important for studying the conditions of carbonate minerals formation in analyzed area of Germanic Basin sediments and also mineral phases stability and solubility^5–13^. The sources of magnesium are usually sea waters and sometimes fresh waters. Magnesium could also come from weathering land carbonate or silicate rocks. After delivering Mg to sea water in a shelf zone dolomite phases sometimes high-Mg calcite are formed^17–20^. Stability of carbonate phase which include Mg ions is usually connected with different cationic size of Ca and Mg, the length of ionic radius and the strength of ionic bonds. The strength of ionic bond of two Ca ions is higher than the strength of ionic bond between Ca and Mg ions^21–24^. So as the crystals of carbonates with Mg substitutions (high-Mg calcites, high magnesium calcites) present weaker stability than the calcite without substitution, stoichiometric dolomite or huntite^25–39^. In low-Mg calcite (low magnesium calcite), the content of Mg varies generally from 0.00 to 3.00%, and the content of Ca from 37.04 to 40.04%. Therefore, the content of MgCO_3_ in low-Mg calcite varies from 0.00 to 10.05%. The content of Mg in high-Mg calcite varies generally from 7.00 to about 11.00% and the content of Ca from 29.04 to 33.04%^9,12^. High-Mg calcite is unstable phase with respect to low-Mg calcite^21–39^. It may lose its magnesium and alter to low-Mg calcite^23–27^. If it is exposed to Mg-rich pore waters, it can gain Mg and be replaced by dolomite^22^. Calcite phase with content of 1.9 mol% of MgCO_3_ is stable compared with both low-Mg calcite and aragonite in temperatures 25 to 64 °C. High-Mg calcite containing up to 15 mol% of MgCO_3_, is stable relative to low-Mg calcite at temperatures greater than 42 °C (up to 60 °C)^21^. The substitution of Mg affects also solubility of calcite phases. It rises with increase of MgCO_3_^20^. High-Mg calcite containing up to 40% of MgCO_3_^40^ is often observed in many natural low-temperature environments^23,24,41^. In dolomite phases and huntite magnesium does not substitute calcium^6,7,9^. It is the chemical element which builds together with Ca, in established proportion, these carbonate phases. So as the dolomite phase and huntite are stable carbonate phases. Dolomite formation depends on Mg/Ca ratio, temperature, CO_2_ content and reaction time^18–20,42^. Non-stoichiometric, poorly ordered proto-dolomite^19,22,42^, is formed during sediment compaction at early stages of diagenesis. Stoichiometric in chemical composition (13.18% of Mg, 46.13% of MgCO_3_) ordered dolomite is formed during advanced stages of diagenesis, in water environment rich in magnesium^18,19,42^. Huntite (20.65% of Mg, 71.92% of MgCO_3_) is usually formed as an effect of hydrothermal processes, weathering of dolomite, or as a result of the transformation of magnesium calcite under high temperature conditions. In sedimentary rocks, it occurs in the sediments of the vadose zone^17,27–29^.

## Materials and methods

**Figure 1:**
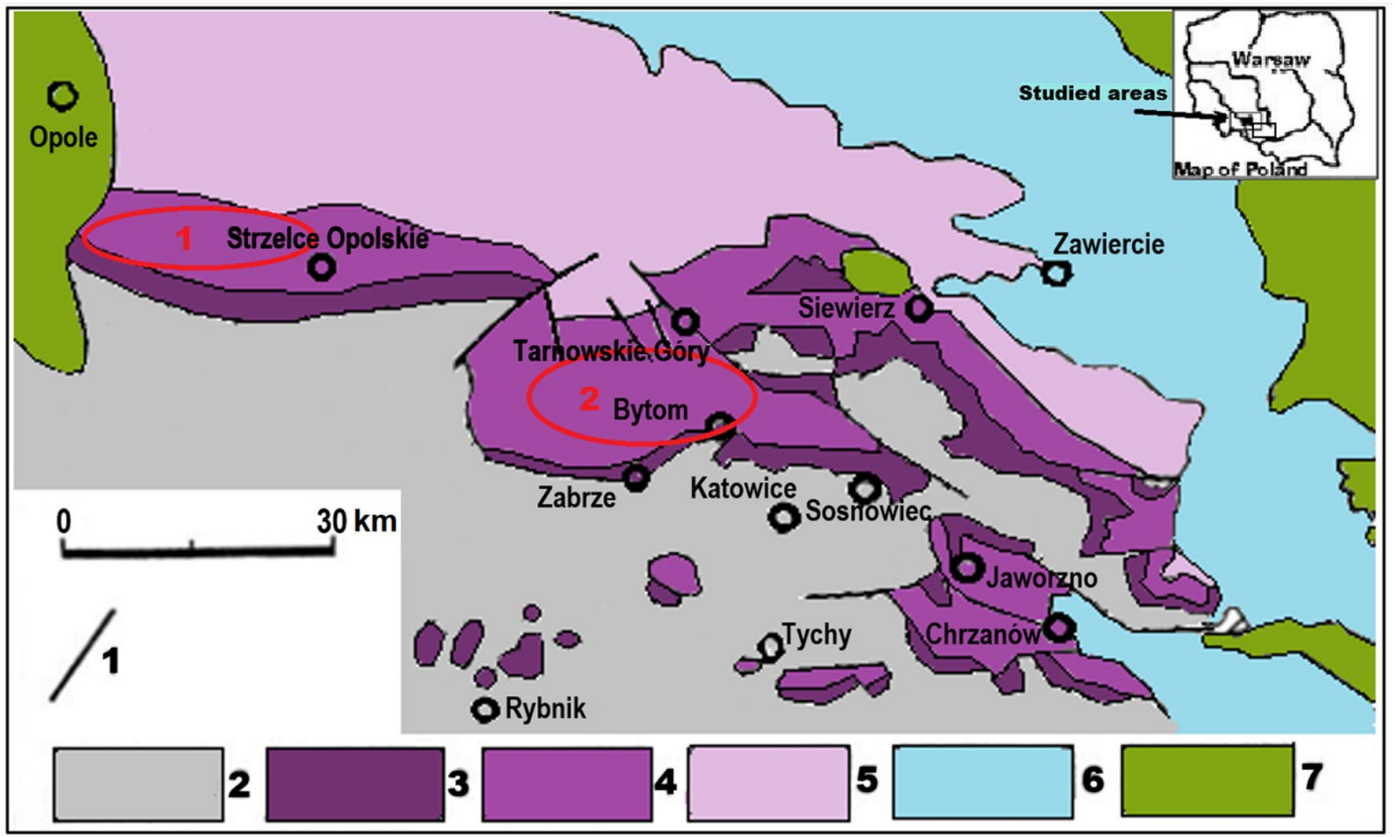
Geological map of the study area with location of the sampling zones 1—more important dislocations; 2—Paleozoic; 3—Bundsandstein; 4—Muschelkalk; 5—Upper Triassic; 6—Jurassic; 7—Cretaceous;  sampling zone. To prepare the map the Corel PHOTO-PAINT Home & Student Suite X6 made in 2012 was used. Build No: 16.1.0.843; Source ID: 807001; DCDHSX6MLEU01. https://www.coreldraw.com/en/pages/coreldraw-x6/.

The limestone samples were collected in the quarries of the Opole Silesia: in Szymiszów (sample S2), in Tarnów Opolski (samples TO7, TO62), in Strzelce Opolskie (sample SO14), dolomites—in Lazarówka Quarry (area of Bytom—sample LZ1) and Piekary Śląskie (sample PSK2) (Fig. 1). 4 limestone samples were studied: S2 from Terebratula Unit and TO7, TO62, SO14 from Karchowice Unit^18^. 2 dolomite samples LZ1, PKS2 from Tarnowice Unit were studied.

X-Ray Diffraction and Electron microprobe analysis were used for identification of mineral phases. X-ray diffraction of limestones was carried out at the Department of Applied Geology in Gliwice using diffractometer HZG4 with a copper lamp and a nickel screen and the following analysis conditions: voltage 35 kV, intensity 18 mA. The method of reflective light was used here. Measurements were executed with a range of d-spacing (Å) from 0.8563 to 0.0953 nm. Carbonate phases in dolomites were identified by X-ray diffraction (XRD) carried out in the laboratory of the Solid State Department, Institute of Physics, University of Silesia using the EMPIRIAN diffractometer by PANALITYCAL. X-ray microanalysis was carried out at the Institute of Non-ferrous Metals in Gliwice. The analyses were conducted using the techniques of X-ray microanalysis EPMA, with application of a JXA-8230 X-ray microanalyser manufactured by JOEL. The examinations were performed on polished sections which were sputtered with a carbon coat. The analysis with the application of WDS spectrometers was carried out in micro-areas of all samples. The WDS method was applied to conduct quantitative analyses in micro-areas, in selected points having different chemical compositions. The content of the following chemical elements was determined: Mg, Si, Al, Ca, Ba, Sr, Fe, Mn, as well as the content of O and C.

## Results

### Results of X-ray diffraction

**Figure 2:**
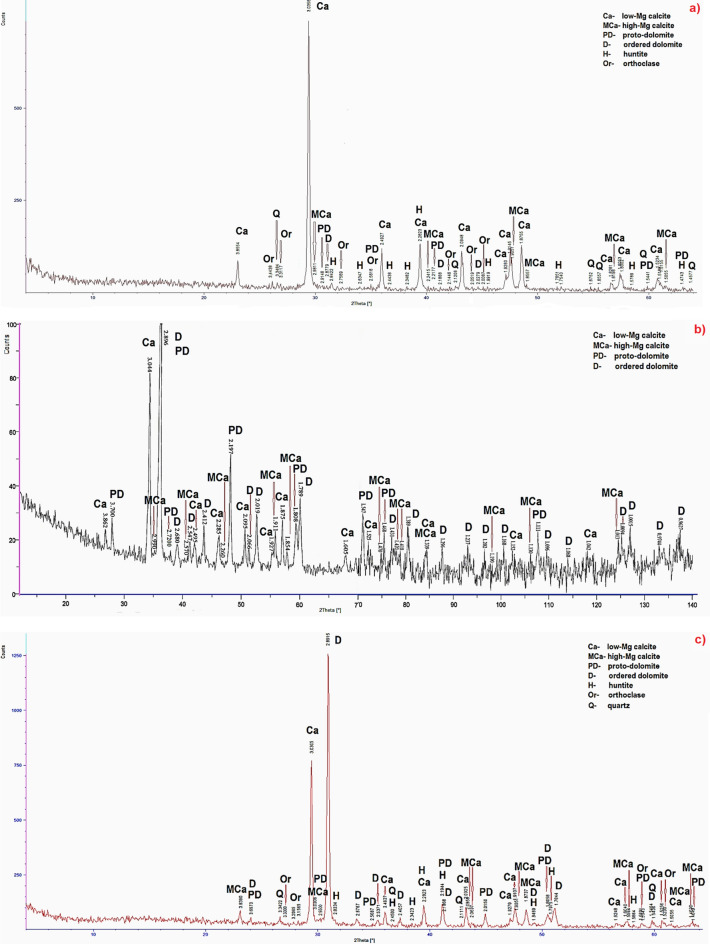

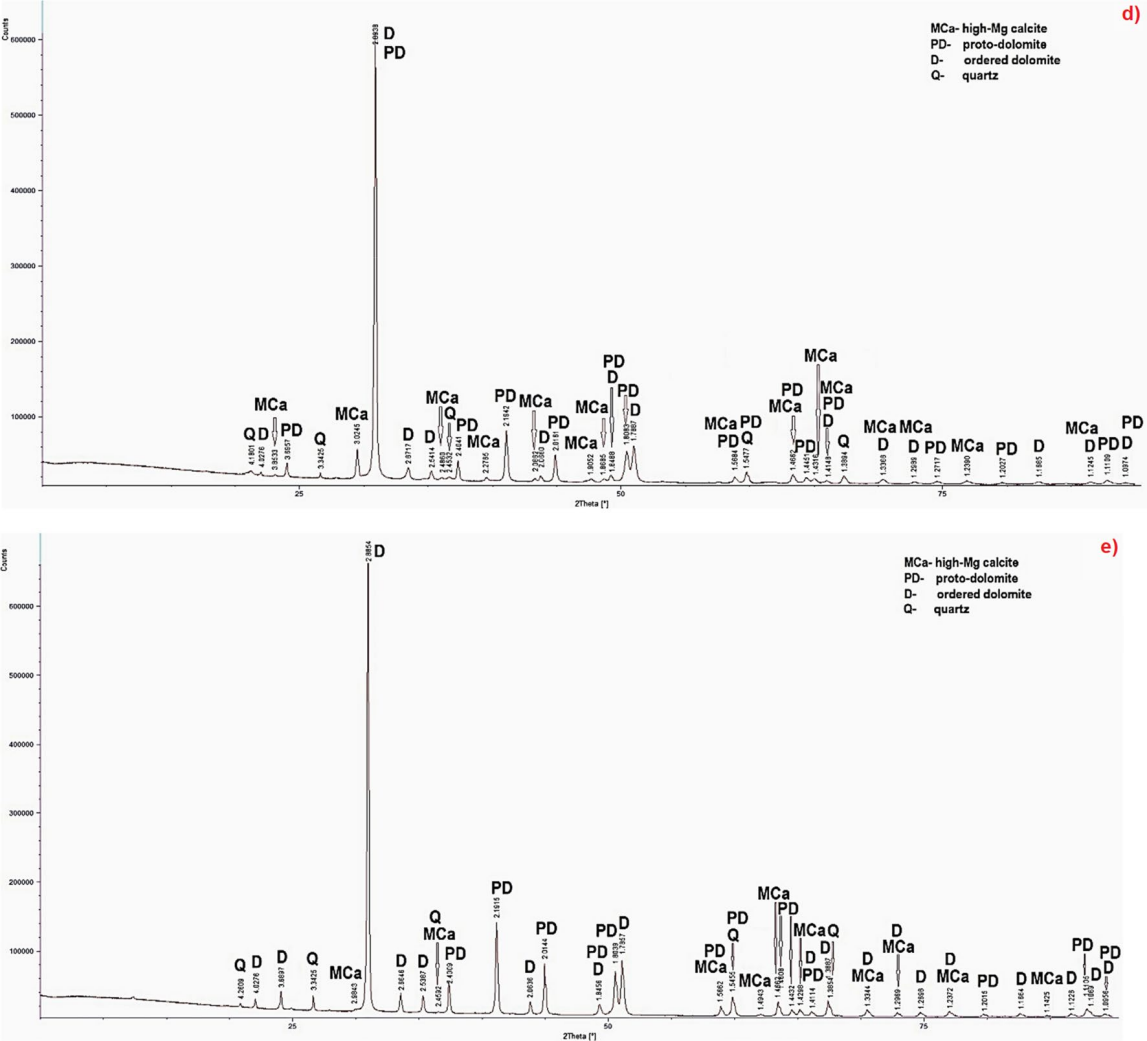
X-ray diffraction patterns of the samples: (**a**) S2, (**b**) TO62, (**c**) SO14, (**d**) LZ1, (**e**) PSK2.

The results of X-Ray Diffraction were in Fig. 2. The following minerals were identified in limestones: low-Mg calcite, high-Mg calcite, proto-dolomite, ordered dolomite, huntite, quartz and orthoclase. In dolomites proto-dolomite, ordered dolomite, high-Mg calcite and quartz were determined.

According to the results generally low-Mg calcite dominates in limestones (Fig. 2a–c). There are also higher amounts of high-Mg calcite, proto-dolomite and ordered dolomite. In samples S2 and SO14 huntite was identified (Fig. 2a,c). Small contents of quartz occur in sample SO14 (Fig. 2c) and orthoclase in samples S2 and SO14 (Fig. 2a,c). The results of X-ray diffraction indicate that sample S2 is typical limestone but the samples TO62 and SO14 are probably dolomitic limestones. In dolomite samples proto-dolomite and ordered dolomite dominate (Fig. 2d,e). There are also higher contents of high-Mg calcite and small amounts of quartz. Therefore these rocks are typical dolomites.

### Results of electron microprobe analysis

**Figure 3:**
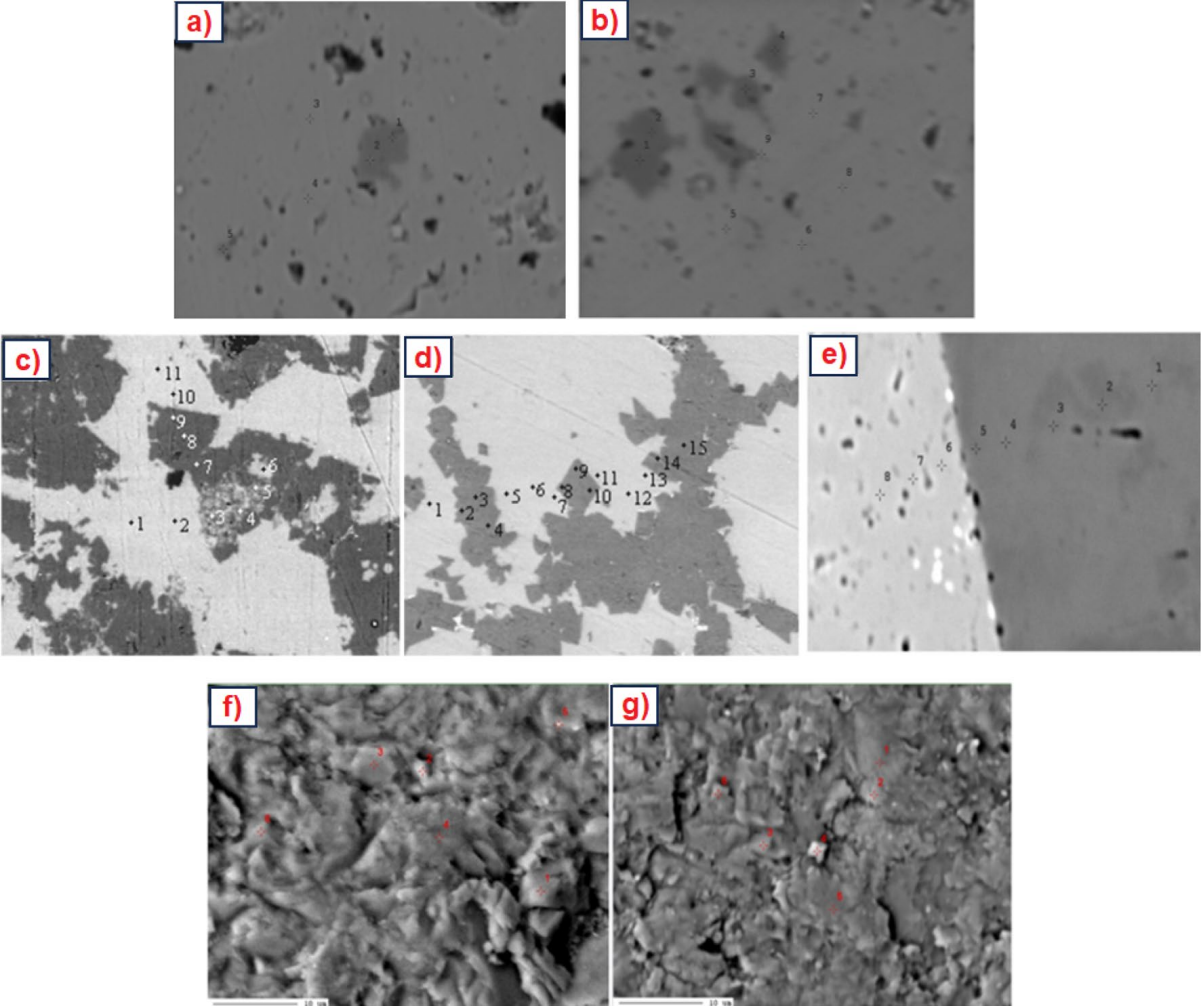
BSE images of the samples: (**a**) S2, first micro-area^6^, (**b**) S2, second micro-area^6^, Magn. ×2000. 1–5—points of chemical analysis; (**c**) TO7, Magn. ×1500, 1–11—points of chemical analysis^5^; (**d**) TO62, Magn. ×1500, 1–15—points of chemical analysis^5^; (**e**) SO14, Magn. ×2000, 1–8—points of chemical analysis^6^; (**f**) LZ1, (**g**) PSK2, Magn. ×2500, 1–6—points of chemical analysis.

The microprobe measurements were executed in points of rocks’ groundmass (Fig. 3, Tables 1, 2, 3, 4). The mineral names in the tables are presented in the form of designations: Ca—low-Mg calcite, MCa—high-Mg calcite, PD—proto-dolomite, D—ordered dolomite, H—huntite. The measurements were made in two micro-areas of sample S2 (Terebratula Unit) (Fig. 3a,b, Table 1). Four carbonate phases were determined: low-Mg calcite, high-Mg calcite, ordered dolomite and huntite. Three samples of Karchowice Unit were studied: TO7^10^, TO62^10^, SO14^11,12^ (Fig. 3c–e, Tables 2, 3). Five carbonate phases were determined: low-Mg calcite, high-Mg calcite, proto-dolomite, ordered dolomite (Fig. 3, Table 2) and huntite (Fig. 3g, Table 3). Two dolomite samples of Tarnowice Beds were studied: LZ1 and PSK2. Only high-Mg calcite and proto-dolomite were determined in these samples (Fig. 3f,g; Table 4). On the basis of the results chemical formulas of identified carbonate phases were calculate. They were presented in Tables from Tables 1, 2, 3, 4.

**Table 1:** Microprobe chemical analyses in the micro-areas of sample S2^6^.

Point number/mineral chemical formula	First micro-area—type of chemical element [%mass] (Fig. 3a)										Total	MgO
	O normalized	C normalized	Mg	Si	Al	Ca	Ba	Sr	Fe	Mn		
1. D [Ca_0.58_,Mg_0.42_CO_3_]	53.80	8.80	13.20	0.00	0.00	24.20	0.00	0.00	0.00	0.00	100.00	22.00
2. MCa (Ca_0.66_,Mg_0.34_)CO_3_	53.80	9.00	10.70	0.00	0.00	26.30	0.00	0.00	0.20	0.00	100.00	17.83
3. Ca (Ca_0.99_,Mg_0.01_)CO_3_	50.00	8.60	0.30	0.00	0.00	41.00	0.00	0.10	0.00	0.00	100.00	0.50
4. Ca (Ca_0.99_,Mg_0.01_)CO_3_	46.70	11.60	0.20	0.00	0.00	41.50	0.00	0.00	0.00	0.00	100.00	0.33
5. MCa (Ca_0.77_,Mg_0.23_)CO_3_	45.90	8.60	7.10	0.00	0.00	38.40	0.00	0.00	0.00	0.00	100.00	11.83
Second micro-area—type of chemical element [%mass] (Fig. 3b)												
1. H [Ca_0.41_,Mg_0.59_CO_3_]	48.37	10.85	16.18	0.00	0.04	23.07	0.84	0.00	0.64	0.00	100.00	26.97
2. H [Ca_0.46_,Mg_0.54_CO_3_]	49.70	10.36	14.85	0.00	0.04	24.24	0.01	0.02	0.75	0.01	100.00	24.75
3. H [Ca_0.44_,Mg_0.56_CO_3_]	50.31	8.22	15.36	0.00	0.03	25.79	0.00	0.01	0.24	0.00	100.00	25.60
4. H [Ca_0.47_,Mg_0.53_CO_3_]	46.14	8.81	14.56	0.00	0.02	23.54	5.69	0.00	0.17	0.03	100.00	24.27
5. Ca Ca_0.99_,Mg_0.01_)CO_3_	45.66	8.66	0.42	0.00	0.00	45.15	0.02	0.00	0.02	0.04	100.00	0.70
6. Ca (Ca_0.99_,Mg_0.01_)CO_3_	48.98	8.58	0.21	0.00	0.02	42.14	0.00	0.00	0.01	0.00	100.00	0.35
7. Ca (Ca_0.99_,Mg_0.01_)CO_3_	44.74	11.07	0.27	0.00	0.00	43.84	0.01	0.00	0.03	0.00	100.00	0.45
8. Ca CaCO_3_	45.46	8.75	0.06	0.00	0.01	45.68	0.00	0.03	0.01	0.00	100.00	0.10
9. Ca (Ca_0.99_,Mg_0.01_)CO_3_	43.46	9.80	3.49	0.00	0.01	43.14	0.01	0.00	0.06	0.01	100.00	5.82

**Table 2:** Microprobe chemical analyses in the micro-area of samples TO7^5^ and TO62^5^.

Point number/mineral chemical formula	Micro-area of sample TO7—type of chemical element [%mass] (Fig. 3c)										Total	MgO
	O normalized	C normalized	Mg	Si	Al	Ca	Ba	Sr	Fe	Mn		
1. Ca (Ca_0.99_,Mg_0.01_)CO_3_	52.39	4.31	0.29	0.09	0.00	42.56	0.00	0.00	0.14	0.00	100.00	0.48
2. Ca [Ca_0.53_,Mg_0.47_CO_3_]	49.45	8.72	0.44	0.07	0.00	41.21	0.00	0.00	0.01	0.03	100.00	0.73
3. MCa (Ca_0.79_,Mg_0.21_)CO_3_	48.70	7.80	6.73	0.15	0.00	36.06	0.00	0.00	0.61	0.01	100.00	11.22
4. MCa (Ca_0.70_,Mg_0.30_)CO_3_	49.51	9.14	9.48	0.71	0.00	30.56	0.00	0.00	0.72	0.03	100.00	15.80
5. MCa (Ca_0.69_,Mg_0.31_)CO_3_	50.41	8. 51	9.80	0.24	0.00	30.35	0.00	0.00	0.82	0.05	100.00	16.33
6. Ca (Ca_0.99_,Mg_0.01_)CO_3_	47.20	9.09	2.44	0.13	0.00	39.54	0.00	0.00	1.14	0.09	100.00	4.07
7. D [Ca_0.57_,Mg_0.43_CO_3_]	52.31	7.76	13.56	0.08	0.00	25.09	0.00	0.00	0.50	0.00	100.00	22.60
8. D [Ca_0.58_,Mg_0.42_CO_3_]	52.83	7.49	13.38	0.08	0.00	24.68	0.00	0.00	0.80	0.01	100.00	22.30
9. D [Ca_0.56_,Mg_0.44_CO_3_]	52.64	7.70	13.67	0.12	0.00	25.06	0.00	0.00	0.76	0.13	100.00	22.78
10. Ca (Ca_0.99_,Mg_0.01_)CO_3_	49.67	8.43	0.35	0.04	0.00	41.60	0.00	0.00	0.13	0.02	100.00	0.58
11. Ca (Ca_0.99_,Mg_0.01_)CO_3_	50.01	8.80	0.32	0.07	0.00	41.01	0.00	0.00	0.03	0.01	100.00	0.53
Micro-area of sample TO62—type of chemical element [%mass] (Fig. 3d)												
1. Ca (Ca_0.99_,Mg_0.01_)CO_3_	51.13	8.21	0.46	0.09	0.00	39.75	0.00	0.00	0.05	0.01	100.00	0.77
2. PD [Ca_0.61_,Mg_0.39_CO_3_]	56.13	8.66	12.34	0.11	0.00	22.40	0.00	0.00	0.47	0.04	100.00	20.57
3. D [Ca_0.57_,Mg_0.43_CO_3_]	47.94	10.31	13.35	0.11	0.00	26.99	0.00	0.00	0.30	0.04	100.00	22.25
4. PD [Ca_0.61_,Mg_0.39_CO_3_]	56.52	8.96	12.23	0.17	0.00	22.27	0.00	0.00	0.46	0,00	100.00	20.38
5. Ca (Ca_0.99_,Mg_0.01_)CO_3_	51.53	7.09	0.44	0.05	0.00	41.08	0.00	0.00	0.00	0,01	100.00	0.73
6. Ca (Ca_0.99_,Mg_0.01_)CO_3_	43.13	9.00	0.20	0.04	0.00	47.44	0.00	0.00	0.00	0.00	100.00	0.33
7. Ca (Ca_0.99_,Mg_0.01_)CO_3_	43.01	8.92	0.16	0.07	0.00	47.59	0.00	0.00	0.02	0.01	100.00	0.27
8. PD [Ca_0.53_,Mg_0.47_CO_3_]	55.91	8.50	12.61	0.05	0.00	22.54	0.00	0.00	0.31	0.02	100.00	21.02
9. PD [Ca_0.60_,Mg_0.40_CO_3_]	56.49	8.03	12.51	0.11	0.00	22.31	0.00	0.00	0.43	0.02	100.00	20.85
10. PD [Ca_0.60_,Mg_0.40_CO_3_]	56.66	7.32	12.53	0.09	0.00	23.02	0.00	0.00	0.12	0.00	100.00	20.88
11. Ca (Ca_0.99_,Mg_0.01_)CO_3_	51.69	7.25	0.21	0.05	0.00	40.66	0.00	0.00	0.06	0.01	100.00	0.35
12. Ca (Ca_0.99_,Mg_0.01_)CO_3_	51.93	7.69	0.12	0.06	0.00	39.88	0.00	0.00	0.05	0.05	100.00	0.20
13. Ca (Ca_0.99_,Mg_0.01_)CO_3_	44.14	9.50	1.08	0.09	0.00	44.94	0.00	0.00	0.12	0.00	100.00	1.80
14. PD [Ca_0.62_,Mg_0.38_CO_3_]	56.31	8.75	11.85	0.08	0.00	22.69	0.00	0.00	0.32	0.01	100.00	19.75
15. PD [Ca_0.62_,Mg_0.38_CO_3_]	56.31	8.75	11.85	0.08	0.00	22.69	0.00	0.00	0.32	0.01	100.00	19.75

**Table 3:** Microprobe chemical analyses in the micro-area of sample SO14^6^.

Point number/mineral chemical formula	Type of chemical element [%mass] (Fig. 3e)										Total	MgO
	O normalized	C normalized	Mg	Si	Al	Ca	Ba	Sr	Fe	Mn		
1. H [Ca_0.49_,Mg_0.51_CO_3_]	48.37	11.54	14.04	0.00	0.03	25.19	0.00	0.02	0.81	0.00	100.00	23.40
2. H [Ca_0.49_,Mg_0.51_CO_3_]	47.32	12.90	14.01	0.00	0.02	24.78	0.00	0.00	0.97	0.00	100.00	23.35
3. D [Ca_0.57_,Mg_0.43_CO_3_]	49.03	11.53	13.42	0.00	0.02	24.87	0.00	0.00	1.09	0.04	100.00	22.37
4. H [Ca_0.47_,Mg_0.53_CO_3_]	46.65	12.19	14.72	0.00	0.03	24.99	0.08	0.00	1.34	0.00	100.00	24.53
5. H [Ca_0.42_,Mg_0.58_CO_3_]	45.60	12.35	15.92	0.01	0.02	24.78	0.00	0.02	1.27	0.03	100.00	26.53
6. Ca (Ca_0.99_,Mg_0.01_)CO_3_	42.81	14.97	0.17	0.00	0.02	41.88	0.00	0.00	0.11	0.02	100.00	0.28
7. Ca (Ca_0.99_,Mg_0.01_)CO_3_	44.71	12.49	0.16	0.00	0.00	42.53	0.00	0.00	0.11	0.00	100.00	0.27
8. Ca CaCO_3_	43.18	12.44	0.10	0.00	0.00	44.02	0.01	0.00	0.24	0.01	100.00	0.17

**Table 4:** Microprobe chemical analyses in the micro-areas of samples LZ1 and PSK2.

Point number/mineral chemical formula	Micro-area of sample LZ1—type of chemical element [%mass] (Fig. 3f)										Total	MgO
	O normalized	C normalized	Mg	Si	Al	Ca	Ba	Sr	Fe	Mn		
1. MCa (Ca_0.71_,Mg_0.29_)CO_3_	54.77	11.99	8.97	0.01	0.07	21.74	0.00	0.00	2.14	0.31	100.00	14.87
2. MCa (Ca_0.73_,Mg_0.27_)CO_3_	54.04	12.02	8.09	0.10	0.08	21.50	0.00	0.00	3.58	0.59	100.00	13.41
3. PD [Ca_0.62_,Mg_0.36_CO_3_]	54.18	11.74	11.12	0.00	0.04	19.72	0.00	0.00	2.82	0.38	100.00	18.44
4. MCa (Ca_0.74_,Mg_0.26_)CO_3_	55.54	11.23	7.35	0.36	0.04	21.00	0.00	0.00	4.01	0.47	100.00	12.19
5. MCa (Ca_0.74_,Mg_0.26_)CO_3_	56.58	11.84	7.72	0.12	0.02	21.78	0.00	0.00	1.70	0.24	100.00	12.80
6. MCa (Ca_0.76_,Mg_0.24_)CO_3_	55.80	11.68	6.69	0.02	0.03	21.59	0.00	0.00	3.75	0.44	100.00	11.09
Micro-area of sample PSK2—type of chemical element [%mass] (Fig. 3g)												
1. MCa (Ca_0.68_,Mg_0.32_)CO_3_	55.45	11.43	9.75	2.10	0.48	20.50	0.00	0.00	0.23	0.06	100.00	16.17
2. MCa (Ca_0.69_,Mg_0.31_)CO_3_	52.10	10.35	9.43	6.68	0.43	20.59	0.00	0.00	0.38	0.04	100.00	15.63
3. PD [Ca_0.66_,Mg_0.34_CO_3_]	54.07	11.53	11.44	0.45	0.15	21.92	0.00	0.00	0.40	0.04	100.00	18.97
4. PD [Ca_0.63_Mg_0.37_CO_3_]	52.08	10.30	12.98	1.84	0.16	22.47	0.00	0.00	0.14	0.03	100.00	21.52
5. MCa (Ca_0.73_,Mg_0.27_)CO_3_	53.74	11.55	8.23	3.07	0.35	22.79	0.00	0.00	0.21	0.06	100.00	13.65
6. PD [Ca_0.64_,Mg_0.36_CO_3_]	54.31	11.18	12.25	0.22	0.11	21.88	0.00	0.00	0.03	0.02	100.00	20.31

Due to the diversity of the sample S2^6^ (Terebratula Unit), microprobe measurements were made in two micro-areas of this sample. The research of the results indicate that the content of MgO in huntite in this limestone is lower than the stoichiometric value for this carbonate phase (MgO—34.25%) (Fig. 3a,b, Table 1). Probably some magnesium was removed from crystals during dehuntization processes. Also thermal decomposition of huntite into aragonite is possible. In one sample of Karchowice Unit—TO7^5^ two calcite phases were identified: low-Mg calcite and high-Mg calcite. Moreover ordered dolomite was determined (Fig. 3c, Tables 2). In samples TO62^5^, SO14^6,8^ (Fig. 3d,e, Table 2) low-Mg calcite dominate. Moreover ordered dolomite was identified and, in sample TO62^10^—proto-dolomite (Fig. 3d, Tables 2), but in sample SO14^6,8^ (Fig. 3e, Table 3)—huntite. As in sample S2, also in sample SO14 huntite is characterized by a reduced content of magnesium in relation to the stoichiometric value for this mineral phase (MgO—34.25%) (Fig. 3e, Table 3).

Slight variation in the amount of mineral phases was observed in the dolomite samples of Tarnowice Unit. Only high-Mg calcite and proto-dolomite were determined in samples LZ1 and PSK2 (Fig. 3f,g, Table 4). Variable Mg content in high-Mg calcite and lowered MgO amount in proto-dolomite were observed. This may indicate that they could be calcareous dolomites.

## Summary of research results

The results of X-ray diffraction and Electron microprobe analysis indicate the presence of five carbonate phases varied in Mg content: low-Mg calcite, high-Mg calcite, proto-dolomite, ordered dolomite and huntite.

The results of X-Ray diffraction indicate that with the increase of magnesium in the crystal the values of diffraction lines decrease^43,44^. Summarizing the results of X-ray diffraction it can be concluded that sample S2 is typical limestone and the samples TO62 and SO14 are dolomitic limestones. The samples LZ1 and PSK2 are typical dolomites.

In BSE images low-Mg calcite and high-Mg calcite are mixed in limestone rock mass (Fig. 3e,f). The content of Mg in low-Mg calcite is below 3% (MgCO_3_ amount is below 10.75%). The content of Mg in high-Mg calcite ranges from 6.69 to 10.70% (MgCO_3_ value ranges from 23.84 to 38.34%). Proto-dolomite is characterized by Mg content from 11.12 to 12.61% (MgCO_3_ value ranges from 39.65 to 45.18%). In ordered dolomite Mg content ranges from 13.02 to 13.67%. (MgCO_3_ value ranges from 46.65 to 48.98%). Therefore in proto-dolomite the MgCO_3_ value is lower than the stoichiometric one for this carbonate phase. In ordered dolomite is similar to stoichiometric. Mg content in huntite ranges from 14.01 to 16.18% and MgCO_3_ value ranges from 50.20 to 57.98%. So as it is lower than stoichiometric value for this carbonate phase which varies from 69.30 to 72.28% of MgCO_3_. The reduction of Mg in huntite could be an effect of diagenetic processes (dehuntization/calcitization?)^6,7,9^ or thermal decomposition of huntite into aragonite^45^.

The results of microprobe measurements indicate that samples S2, TO7, TO62 and SO14 represent dolomitic limestones with different content of carbonate phases varied in Mg amount. The samples LZ1 and PSK2 represent typical dolomites including proto-dolomite and high-Mg calcite.

## Discussion

In the Triassic (Lower Muschelkalk) limestones from the area of Opole Silesia, five carbonate phases were identified: low-Mg calcite, high-Mg calcite, proto-dolomite, ordered dolomite and huntite. Two carbonate phases were identified in the Triassic (Upper Muschelkalk) dolomites from the area of Upper Silesia: high-Mg calcite and proto-dolomite.

On the basis of the results the chemical formulas of identified carbonate phases were calculated and probable crystal structures of these minerals were created. The results were compared with the data obtained by other scientists presented in their works^6,29,35,45–53^. The crystal structures of the carbonate phases were prepared on the basis of data from references^6,29,35,45–48^. Preparing the frameworks of crystal structures, the Mg content shown in the calculated chemical formulas was included. The research results allow to determine cell parameters of identified carbonate phases including the data from previous studies^26,35^ (Table 5). It was also possible to determine the appropriate space group of identified mineral phases comparing the obtained data with information from the studies of other scientists^26,35^ (Table 5).

**Table 5:** Structures of crystal cell and chemical formulas of carbonate phases with magnesium.

No	Carbonate phase name	Chemical formula	Cell parameters^26,35^	Space group^26,35^
1	Low-Mg calcite Fig. 4a	(Ca_1.00–0.95_,Mg_0-0.05_)CO_3_	a_o_ = 4.989 Å, c_o_ = 17.062 Å	Scalenohedral—R$\overline{3}$c
2	High-Mg calcite Fig. 4b	Gogolin unit Ca_0.9_Mg_0.1_CO_3_ Górażdże unit (Ca_0.92–0.90_,Mg_0.08–0.10_)CO_3_ Terebratula unit (Ca_0.87–0.74_,Mg_0.13–0.26_)CO_3_ Karchowice unit (Ca_0.85–0.77_,Mg_0.15–0.23_)CO_3_ Tarnowice unit (Ca_0.76–0.63_,Mg_0.24–0.37_)CO_3_	a_o_ = 4,941 Å, c_o_ = 16,854 Å	Rhombohedral—R$\overline{3}$c
3	Proto-dolomite Fig. 4c Ordered dolomite Fig. 4d	(Ca,Mg)(CO_3_)_2_ [Ca_0.60–0.62_,Mg_0.40–0.38_CO_3_] (Ca,Mg)(CO_3_)_2_ [Ca_0.5_,Mg_0.5_CO_3_]	a_o_ = 4.842 Å, c_o_ = 15.95 Å	Rhombohedral-proto-dolomite—R$\overline{3}$c Rhombohedral-ordered dolomite—R$\overline{3}$
4	Huntite stoichiometric Fig. 4e(A) De-Huntite Fig. 4e(B)	CaMg_3_[CO_3_]_4_ [Ca_0.25_,Mg_0.75_CO_3_] CaMg_3_[CO_3_]_4_ [Ca_0.42–0.49_,Mg_0.51–0.58_CO_3_]	a_o_ = 9.5027 Å, c_o_ = 7.8212 Å	Trapezohedral—R32

**Figure 4:**
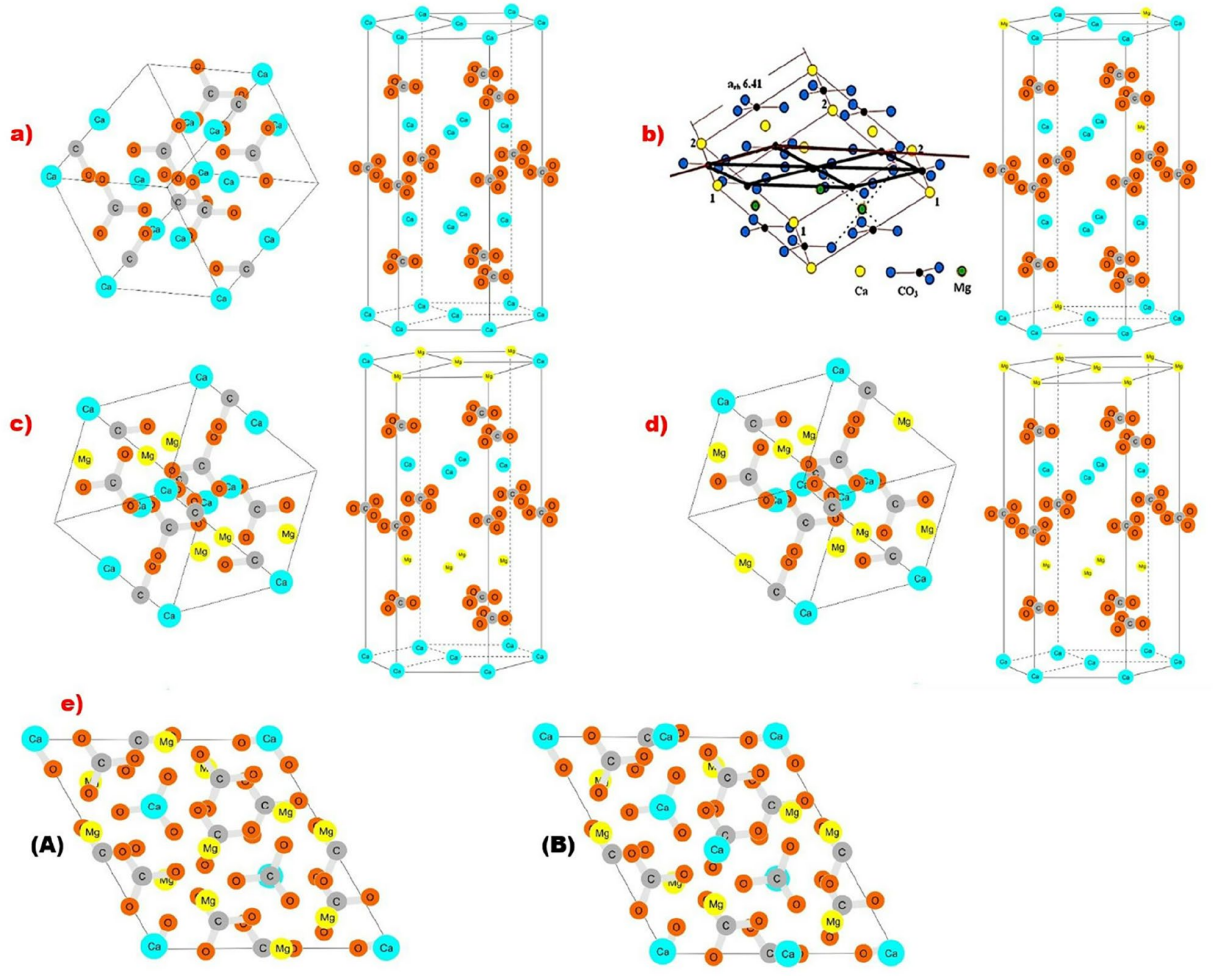
Crystal structures of the carbonate phases: (**a**) low-Mg calcite, (**b**) high-Mg calcite (on the left side- made by Author^6^), (**c**) proto-dolomite, (**d**) ordered dolomite, (**e**) (A) stoichiometric huntite, (B) de-huntite. To prepare the map the Corel DRAW Home & Student Suite X6 made in 2012 was used. Build No: 16.1.0.843; Source ID: 807,001; DCDHSX6MLEU01. https://www.coreldraw.com/en/pages/coreldraw-x6/.

According to the results low-Mg calcite (Fig. 4a, Table 5) was determined in the Lower Muschelkalk limestones but not in Upper Muschelkalk dolomites. High-Mg calcite (Fig. 4b, Table 5) determined in limestones of Lower Muschelkalk and dolomites of Upper Muschelkalk is characterized by differences in Mg content. In a single crystal cell of high-Mg calcite which is built of 14 ions three of them could be Mg ions and 11—Ca ions^5–7,9^. With the increase of MgCO_3_ content in high-Mg calcite the values of cell parameters- a_o_ (Å) and c_o_ (Å) drop^40,54^. If the amount of MgCO_3_ is 22.7% high-Mg calcite present the cell parameters as follows: a = 4.91 Ǻ, c = 16.65 Ǻ and for the amount of MgCO_3_ 36.71% their values are lower: a = 4.88 Ǻ, c = 16.45 Ǻ^40,54^. Magnesium substitutions in high-Mg calcite make the structure of crystal cell different in comparison with low-Mg calcite. This is related to the difference in the size of the ionic radii of Ca and Mg^37,38,54,55^. The symmetry of high-Mg calcite crystal is rhombohedral (space group R3c) similar to the typical for proto-dolomite (Table 5)^26,35^. Two dolomite phases were determined: proto-dolomite and ordered dolomite. Proto-dolomite is characterized by lower content of magnesium than ordered dolomite, but higher than high-Mg calcite. Moreover, the MgO content in proto-dolomite is lower than the stoichiometric value for dolomite (MgO—21.86%, Mg—13.18%). The symmetry of proto-dolomite crystal (Fig. 4c, Table 5) is rhombohedral with the space group R3c^27,36,46^, similar to the typical one for high-Mg calcite. The space group of ordered dolomite (Fig. 4d, Table 5) is rhombohedral—R3 but a little bit different to that of proto-dolomite^27,36^. The content of MgO is similar to stoichiometric value for this carbonate phase. In the Triassic limestones of the Polish part of the Germanic Basin (Opole Silesia) also huntite was identified^6,7,9,47^. However this huntite presents lower value of MgCO_3_ (50.20% to 57.98%) than the typical one for stoichiometric value for this carbonate phase, which ranges from 69.30 to 72.28%. The reduction of Mg in this mineral can be an effect of diagenetic processes—dehuntization (calcitization?)^6,7,9,47^. This phase was named as de-huntite. On the basis of determined chemical formula and crystal structure of huntite with stoichiometric magnesium content (Fig. 4e(A))^29,47^ theoretical structure of de-huntite was drawn up (Fig. 4e(B), Table 5).

According to the results low-Mg calcite and high-Mg calcite were probably formed in the epicontinental Germanic Basin during direct crystallization from sea water, at the same time as aragonite and dolomite phases. Moreover, owing to the presence of waters of the phreatic zone and salty sea waters which had an elevated content of dissolved mineral components, the fixation process of unstable high-Mg calcite originally formed in the environment of the seabed took place^6,7,9^. High-Mg calcite and aragonite are an unstable phases which undergo transition into low-Mg calcite. Only the presence of Sr and Ba in calcite indicate that the primary phase was aragonite. High-Mg calcite was probably preserved in Triassic rocks during early diagenetic processes. Dolomite phases were formed in the mixing zone of the waters from the phreatic zone and salty sea waters in the early stage of constructive diagenesis during dolomitization processes. In this environment proto-dolomite and ordered dolomite could have been formed. However the transition of proto-dolomite into ordered dolomite during advanced stages of diagenesis is possible. Huntite of sedimentary rocks is formed in the vadose zone^6,7,9,17^. De-huntite of the Germanic Basin was probably formed in the areas in which diagenetic processes were taking place with the contribution of waters from the vadose zone. It could be the reason for the reduced content of magnesium in this mineral. Transition process connected with reducing magnesium ions from huntite crystals can be described as dehuntization (calcitization?).

The research results were compared with the others’ works in the context of previous works including world data. It has been done to increase the scientific significance of this article. According to the data from previous studies the content of Mg in low-Mg calcite varies from 0.00 to 3.00%^1,2,9,12,18,19,55^. The research results show that in Triassic limestones the Mg content ranges from 0.06 to 3.49%. These values are therefore comparable to the data obtained by other scientists. The low-Mg calcite crystal structure was prepared on the basis of data presented by Packet and Reader^35^ and Maslen et al.^45^. It is the theoretical structure of a low-Mg calcite crystal with no Mg ions in crystal structure. The content of Mg in high-Mg calcite varies generally from 7.00 to about 11.00%^9,12,18–26,32^. Triassic limestones include high magnesium calcite with amount of Mg from 6.73 to 10.70%. Therefore, the research results are similar to the data from previous studies. In the previous work, a hypothetical structure of magnesium calcite was prepared, in which the arrangement of magnesium ions in the crystal was proposed^6^. It was based on the models proposed by Wenk et al. and Tsipursky and Buseck^6,37,56^. Wenk et al.^56^ found that magnesium calcite has a ν-type structure. In this structure, in addition to layers containing calcium ions, there will also be layers containing Ca and Mg ions along the [100] direction, which gives a calcium to magnesium ratio of 3:1 (Ca:Mg = 3:1)^6,37,56^. According to the information from references^19,22,31,40,42,48^ proto-dolomite is non-stoichiometric, poorly ordered dolomite phase. It is characterized by a reduced Mg content compared to the stoichiometric value of dolomite^18–20,26,31,40^. Proto-dolomite was identified in one limestone sample TO62 (from Karchowice Unit) and in dolomite samples (from Tarnowice Unit). Proto-dolomite of limestone is characterized by Mg content from 11.85 to 12.61%. Mg content in proto-dolomite of dolomites ranges from 11.12 to 12.98%. These values are higher than typical for high-Mg calcite but lower to stoichiometric for dolomite. Proto-dolomite is treated by some scientist as a phase formed from high-magnesium calcite. It is carbonate transitional phase between magnesian calcite and ordered dolomite^31,40,48^. High-Mg calcite is characterized by 0–32 mol% of Mg substitution for Ca. Proto-dolomite has about 55–60 mol% of Ca in the lattice with incomplete segregation of Ca and Mg into separate layers^48^. Stoichiometric, ordered dolomite is characterized by 50:50 of Ca to Mg ratio with the near perfect ordering of the Mg and Ca in alternate cation layers^48^. The proto-dolomite crystal structure was prepared on the basis of the ordered dolomite crystal structure, include the reduced number of magnesium ions determined in proto-dolomite. Ordered dolomite is characterized by stoichiometric value of Mg for this carbonate phase (MgO—21.86%, Mg—13.18%)^18–20,22,26,48,51,52^. Mg content in ordered dolomite of Triassic limestones ranges from 13.20 to 13.67%. Therefore the measured contents are slightly higher than the stoichiometric value for dolomite. The ordered dolomite crystal structure was prepared on the basis of the data presented by Antao et al.^46^ and Mehmood^48^. In the ordered dolomite crystal structure the amount of Ca and Mg ions is the same. Huntite is carbonate phase characterized by higher Mg content (20.65% of Mg, 71.92% of MgCO_3_) than in ordered dolomite^17,27–30^. However, the determined Mg content in huntite of Triassic limestones is definitely lower than stoichiometric one. It ranges from 14.01 to 15.92%. That's why this carbonate phase was named as de-huntite. The reduced content of Mg in this mineral can be an effect of dehuntization^6,7,9,47^. De-huntite crystal structure was prepared on the basis of the structure of huntite presented by Dollase and Reeder in their work^29^. Creating de-huntite crystal structure, a reduced amount of Mg ions was included.

## Conclusions

Five carbonate phases were determined in Triassic limestones and dolomites of Polish Part of Germanic Basin: low-Mg calcite, high-Mg calcite, proto-dolomite, ordered dolomite and de-huntite. Low-Mg calcite and de-huntite occur only in Triassic limestones. In dolomites only high-Mg calcite was identified. Moreover Triassic dolomites are characterized by presence of proto-dolomite and lack of ordered dolomite and de-huntite.

The content of Mg in high-Mg calcite is higher than in low-Mg calcite but lower than in proto-dolomite. Proto-dolomite is characterized by lower Mg content than typical for stoichiometric one for dolomite. Ordered dolomite present Mg content similar to the stoichiometric value. De-huntite presents lower value of MgCO_3_ than typical for stoichiometric one for huntite. The reduction of Mg in de-huntite can be an effect of diagenetic process, dehuntization. On the basis of the results chemical formulas of identified carbonate phases were calculated and crystal structures of these minerals were prepared including study results and reference data. Research results indicate the similarity of the geochemical composition of Triassic limestones from the Opole Silesia and dolomites from Upper Silesia which include carbonate phases with different Mg content with the world data on Triassic carbonate rocks presented in the references.

The data allowed to form the theory about possible formation of identified carbonate phases and diagenetic processes that influenced their current structure and preservation of unstable high-Mg calcite. Low-Mg calcite and high-Mg calcite were formed during direct crystallization from sea water, at the same time as aragonite and dolomites. High-Mg calcite was preserved in Triassic rocks during diagenetic processes. Owing to the presence of the phreatic zone waters and salty sea waters also the fixation process of high-Mg calcite could take place. Dolomite phases were formed in the mixing zone of the waters from the phreatic zone and salty sea waters during dolomitization processes. But the transition of proto-dolomite into ordered dolomite during advanced stages of diagenesis is possible. De-huntite was formed in the areas of Germanic Basin where diagenetic processes were taking place with the contribution of waters from the vadose zone.

To obtain new data on carbonate phases with different Mg content, the Triassic limestones of Opole Silesia and the Triassic dolomites of Upper Silesia will be subjected to further analyses. Especially two methods will be used: X-Ray Fluorescence (XRF) and Fourier Transform Infrared Spectroscopy (FTiR). They will provide new data on the presence of carbonate phases with different Mg amount in the studied Triassic rocks and the range of Mg content in these phases.

## Data Availability

The datasets used and analysed during the current study are available from the corresponding author on reasonable request.
